# Optimization study of 2-hydroxyquinoxaline (2-HQ) biodegradation by *Ochrobactrum* sp. HQ1

**DOI:** 10.1007/s13205-015-0358-6

**Published:** 2016-02-08

**Authors:** G. V. Subba Reddy, M. Md. Rafi, S. Rubesh Kumar, N. Khayalethu, D. Muralidhara Rao, B. Manjunatha, G. H. Philip, B. R. Reddy

**Affiliations:** 1 Department of Microbiology, Sri Krishnadevaraya University, Anantapuramu-, 515003 Andhra Pradesh India; 2 Department of Agriculture and Animal Health, University of South Africa, Private Bag X6, Florida, 1710, Johannesburg, South Africa; 3 Department of Biotechnology, Sri Krishnadevaraya University, Anantapuramu-, 515003 Andhra Pradesh India; 4 Department of Pharmaceutical Analysis, JNTUA-Oil Technological Research Institute, Anantapuramu-, 515001 Andhra Pradesh India; 5 Department of Life Sciences, Universidad de las Fuerzas Armadas-ESPE, Sangolquí, Quito, Ecuador, South America

**Keywords:** 2-Hydroxyquinoxaline, *Ochrobactrum* sp. HQ1, 16S rRNA sequence analysis, HPLC and GC–MS analysis

## Abstract

A novel aerobic gram-negative bacterial strain capable of utilizing 2-hydroxyquinoxaline (2-HQ) as sole source of carbon and energy was isolated from Indian agricultural soil and named as HQ1. Strain HQ1 was identified as *Ochrobactrum* sp. on the basis of morphology, physico-biochemical characteristics and 16S rRNA sequence analysis. The generation time of *Ochrobactrum* sp. HQ1 on 2-HQ at log phase is 0.71 h or 42.6 min. The degradation of 2-HQ by HQ1 under various physico-chemical parameters was analysed by HPLC and observed to be optimum with a high inoculum density (1.0 OD) at pH 7–8, temperatures 37–40°C and a high concentration of 2-HQ (500 ppm). Degradation of 2-HQ was also improved when additional nitrogen sources were used and this was attributed to the enhanced growth of the bacterium on the readily available nitrogen sources. Analysis of 2-HQ degradation by GC–MS resulted in elucidation of the degradation pathway for HQ1, a novel observation for aerobic Gram-negative bacteria. These findings are a possible indication of the application of HQ1 in the bioremediation of pesticide/metabolite contamination.

**Electronic supplementary material:**

The online version of this article (doi:10.1007/s13205-015-0358-6) contains supplementary material, which is available to authorized users.

## Introduction

Quinalphos is one of the major and most widely used organophosphorus insecticides in agriculture and undergoes microbial metabolism/chemical hydrolysis to form major metabolites—2-hydroxyquinoxaline (2-HQ) and diethyl thiophosphate/diethyl phosphate (Babu et al. 1998; Menon and Gopal 2003; Goncalves et al. 2006; Gupta et al. 2012; Kaur and Sud 2012; Talwar et al. 2014) in soil and water. Diethyl phosphate/diethyl thiophosphate, formed from number of organophosphates including quinalphos, parathion and chlorpyrifos, undergoes rapid mineralization because of it easy utilization by microorganisms as carbon and phosphorus source (Cook et al. 1978). 2-HQ is getting accumulated in environment because of its longer persistence and extensive use of quinalphos (Babu et al. 1998). Accumulation of 2-HQ in the environment poses a health hazard to animals and human because of 2-HQ is capable of destroying various biogenic amines such as serotonin, melatonin, N^1^-acetyl-5-methoxy kynuramine, dopamine, epinephrine and norepinephrine and inducing oxidative stress as reflected by generation of H_2_O_2_ (Hardeland et al. 2000). This metabolite has been observed to display toxicity to even aquatic organisms such as ciliates, dinoflagellates and rotifer (Behrends et al. 2004) and has also been reported to be genotoxic to *Salmonella typhimurium* in Ames test (Riediger et al. 2007). Toxicity is not totally eliminated with degradation of the parent compound quinalphos to 2-HQ in the environment. 2-HQ is least understood in terms of biodegradation among metabolites formed from organophosphates (Singh and Walker 2006; Caceres et al. 2010). As microbial agents involved in the biodegradation of this metabolite in the environment are highly useful in decontamination of polluted environment. The aim of the present study was to isolate bacteria that play a role in the degradation of 2-HQ and evaluate the degradation potential under various environmental factors.

## Materials and methods

### Soil sample

Soil sample [organic matter (%) 0.42; nitrogen (%) 1.54; pH 8.1] was collected from *Chrysanthemum indicum* agriculture field in Pendlimarry village, YSR Kadapa district, Andhra Pradesh, India.

### Chemicals

2-HQ was purchased from Sigma-Aldrich Fluka (99 %). This 2-HQ was used for bacterial growth as sole source of carbon, nitrogen and energy. All other chemicals and solvents used in the present study were of analytical reagent grade/HPLC grade and purchased from Sigma-Aldrich.

### Culture medium and selective enrichment method

The mineral salts medium (MSM) contained (gL^−1^) 1.5 NH_4_NO_3_, 1.5 K_2_HPO_4_·3H_2_O, 0.2 MgSO_4_·7H_2_O, 1.0 NaCl and 1 mL of trace elements stock solution. The trace element stock solution contained (gL^−1^): 2.0 CaCl_2_·2H_2_O, 0.2 MnSO_4_·4H_2_O, 0.1 CuSO_4_·2H_2_O, 0.2 ZnSO_4_·H_2_O, 0.02 FeSO_4_·7H_2_O, 0.09 CoCl_2_·6H_2_O, 0.12 Na_2_MoO_4_·2H_2_O and 0.006 H_3_BO_3_.

The selective enrichment culture technique was set-up by inoculating 100 mL of sterile MSM containing 0.005 % 2-HQ with 5 g of agricultural soil. The Erlenmeyer flask was incubated in an orbital shaker (Orbitek LE-IL Model) at 37°C and 175 rpm. After 5 days of incubation period, 5-mL portion of the culture was transferred to fresh medium with high 2-HQ concentration up to 0.05 % in Erlenmeyer flasks and the flasks were incubated for 5 days. After five more transfers, the culture was purified by serial dilution transfer method and streak plating onto solidified MSM containing 0.005 % of 2-HQ. Finally, a pure bacterial strain was obtained and designated as HQ1.

### Identification and characterization of bacterial strain

#### Morphological and physico-biochemical characterization

Morphological observations of bacterial isolate were made with an optical compound microscope. Physiological and biochemical properties of the strain were determined by the procedures as described by Bergey’s manual of determinative bacteriology (Holt et al. 1994).

### 16S rRNA gene sequencing and phylogenetic tree analysis

The total genomic DNA was extracted from the bacterial isolate (HQ1) following a standard phenolic extraction procedure (Sambrook et al. 1989). Phylogenetic analysis based on 16S rRNA gene sequence was performed as described by Qin et al. (2007). The 16S rRNA gene sequence of strain HQ1 was amplified from the genomic DNA with universal conserved sequence as primers—16 forward primer sequence—5′-AGACTCAGGTTTGATCCTGG-3′ and 16 reverse primer sequence—5′-ACGGCTACCTTGTTACGACTT-3′. Both forward and reverse sequences were generated and combined to get an assembled partial sequence of 16S rRNA gene. The determined sequence was compared to those in the GenBank/EMBL data base using the online BLAST program (Altschul et al. 1990). Sequences of the HQ1 and closely related bacterial spp. were collected, aligned and neighbour-joining and maximum-likelihood tree constructed using the Robust Phylogenetic tree online tool (Dereeper et al. 2008, 2010) to establish the phylogenetic relationship.

### Measurement of bacterial growth kinetics on 2-HQ

For growth of bacterial isolate on 2-HQ, 50 mL of sterile MSM in sterile 250 mL Erlenmeyer flasks was spiked with 2-HQ at concentration of 50 µg mL^−1^. Meanwhile, the HQ1 inoculum was prepared by growing the strain in 50 ml of MSM supplemented with yeast extract (0.1 %) and 50 ppm of 2-HQ per ml of MSM and incubating in an orbital shaker at 175 rpm at 37°C overnight. The overnight culture was harvested aseptically (8000 g, 15 min, 4°C) and thoroughly washed with MSM and suspended in sterile MSM to get suspension with the desired OD. Flasks with test chemical—2-HQ in MSM were inoculated with the bacterial culture to the final OD of 1.0 per mL of MSM. Uninoculated flasks with fortified medium served as control. All flasks were incubated in an orbital shaker at 175 rpm at 37°C for 24 h. Five-millilitre aliquots from growing culture broth were withdrawn at 6 h intervals and the growth monitored at 600 nm in a UV–Visible spectrophotometer (Chemito UV-2600). The total number of viable bacterial colony-forming units was determined by the serial dilution method on nutrient agar medium. The specific growth rate of potential isolate HQ1 was calculated in the logarithmic phase.

### Biodegradation of 2-HQ

Experiments for biodegradation of 2-HQ by the bacterial isolate were carried out in 250 mL Erlenmeyer flasks in the same manner as done for growth experiments (2.5). At regular intervals of 24 h, 10 mL of culture filtrate was aseptically withdrawn for growth measurements in a spectrophotometer. After growth measurements culture/medium in both uninoculated and inoculated flasks was processed for residue analysis and centrifuged at 8000*g* for 15 min in a refrigerated centrifuge (REMI, C24 BL, Hyderabad). Supernatants collected in this fashion were extracted with dichloromethane with equal volume of supernatant; this was repeated three times. The extracts were pooled together, dried over anhydrous sodium sulphate, filtered and allowed to dry at room temperature. The residue was dissolved in methanol for HPLC and GC–MS analysis.

#### Optimization of biodegradation of 2-HQ

Appropriate modifications in growth conditions of the bacterial culture on 2-HQ were made to assess the effect of various factors on degradation of 2-HQ by HQ1. For this purpose, MSM was spiked with 50 mg L^−1^ of 2-HQ at and distributed into 250-mL flasks (100 mL per flask). The flasks were supplemented with an additional carbon source (glucose or sodium acetate) or nitrogen source [NH_4_Cl, (NH_4_)_2_SO_4_, urea or yeast extract] to a final concentration of 0.01 % (w/v). Flasks were inoculated with bacterial suspension to get an initial OD of 1.0 and flasks devoid of inoculums maintained as controls. These were incubated 37°C and 175 rpm in a shaker, samples collected at 48 h intervals and the culture supernatant was subjected to dichloromethane extraction prior to residue analysis. The effect of the concentration of 2-HQ on degradation was assessed by growing the bacterial isolate on MSM supplemented with different concentrations of 2-HQ (50–500 ppm). In another experiment, flasks containing MSM (pH-7) supplemented with 50 mg L^−1^ 2-HQ were inoculated with the bacterial cell suspension to an initial OD of 1.0 and incubated in a shaker at 175 rpm at different temperature values (30–45°C) to study the influence of temperature on the degradation of 2-HQ. In order to study the effect of pH on 2-HQ degradation, HQ1 was cultured as described above and only the pH was varied from pH 5 to pH 9.

### Analytical methods

#### Analysis of 2-HQ residue by high-performance liquid chromatography (HPLC)

Residue of 2-HQ extracted from the different experiments was dissolved in methanol and analysed by HPLC (Shimadzu, Japan) equipped with a ternary gradient pump, programmable variable wavelength UV detector, column oven, electric sample valve ODS-2, and C_18_ reverse-phase column (4.6 × 250 mm × 5 μm). The 2-HQ residue analysis was conducted using an isocratic mobile phase of methanol. Sample injection volume was 20 µL, the mobile phase was programmed at flow rate of 1 mL min^−1^ and 2-HQ was detected at 345 nm wave length under these operating conditions with retention time of 0.833 min.

#### Detection and identification of 2-HQ metabolites by GC–MS analysis

MSM was spiked with 2-HQ at 50 µg mL^−1^ in the same manner as mentioned earlier in “Measurement of bacterial growth kinetics on 2-HQ”. MSM (mL) spiked with 2-HQ was distributed into 250 mL Erlenmeyer flasks—(50 ml per flask). Flasks were divided into two main groups, the inoculated (experiment) and the uninoculated (control). Two sets of flasks were incubated under optimal conditions at 37°C and 175 rpm for 5 days. At regular intervals the metabolites were extracted from 10 mL aliquots of culture with dichloromethane and analyzed in GC-MS-QP-5050 chromatograph (M/s. Shimadzu Instruments, Japan) with the column used as ZB-5 capillary column (25 m × 0.32 mm) supplied by (M/s. J and W Scientific, USA). Toluene was used as external standard for the quantification of the compounds.

## Results and discussion

### Isolation and identification of bacterial isolate HQ1

The bacterial strain (HQ1) was isolated from *Chrysanthemum indicum* agricultural soil by selective enrichment method. The strain was identified according to classification scheme outlined by Bergey’s manual of determinative bacteriology (Holt et al. 1994). The cell morphology for HQ-1 was analysed by compound microscopy and observed to display morphological characteristics that are consistent with Gram-negative bacteria; moreover the colonies were punctiform, translucent and with entire margin. Some biochemical tests were performed with the bacterial strain and recorded as Indole test—Negative; Methyl red test—Positive; VP test—Negative; Citrate utilization test—Positive; Glucose and lactose fermentation tests—Negative; Urease activity—Positive; Catalase activity—Negative; Nitrate-reductase activity—Negative; Starch hydrolysis—Positive; Casein hydrolysis—Negative; Gelatin liquefaction—Positive. Based on this morphological and biochemical characteristics the strain HQ1 is homologous with *Ochrobactrum* sp.

**Fig. 1:**
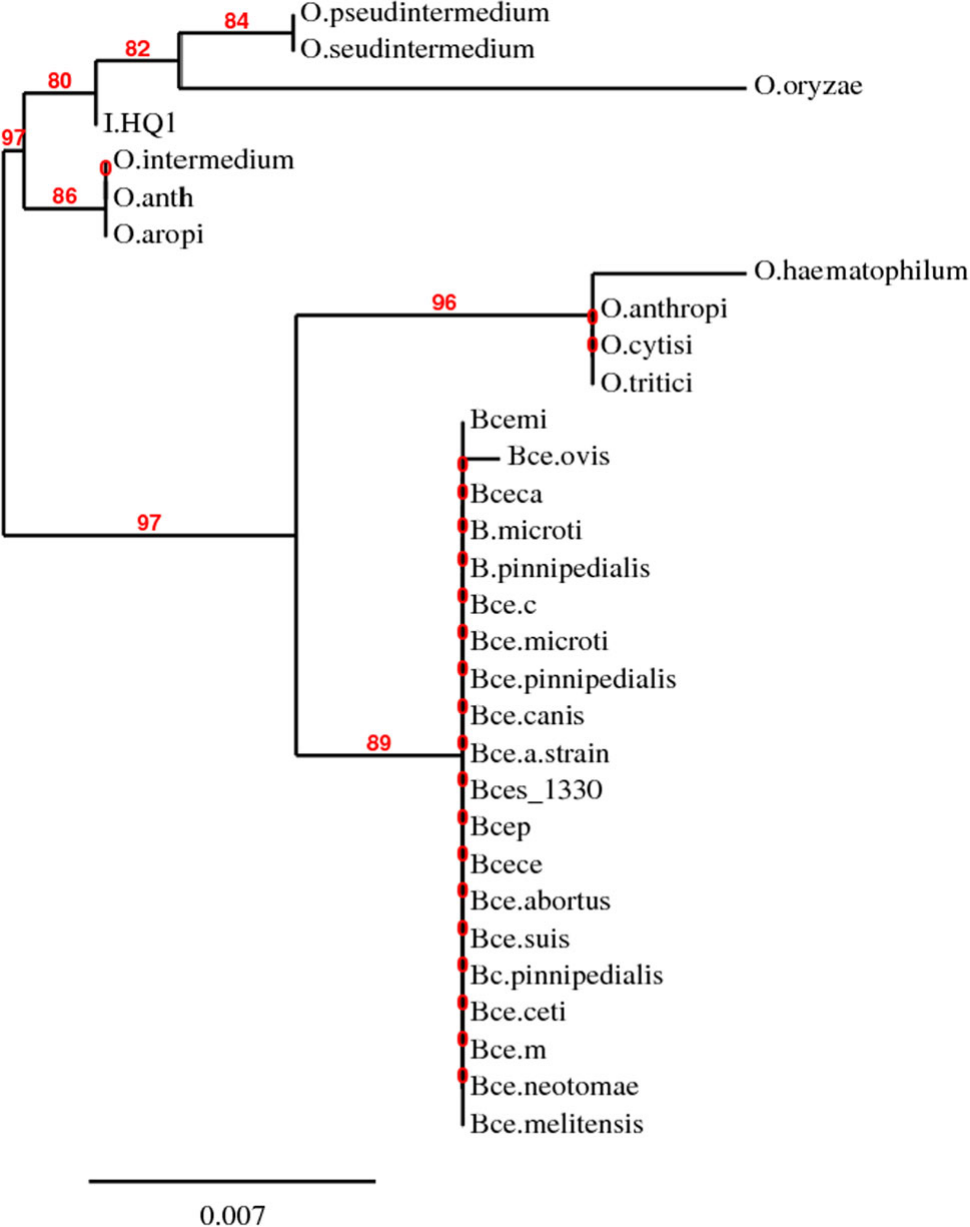
Phylogenetic tree based on the 16S rRNA gene sequences of strain HQ1. Robust Phylogenetic tree showing the phylogenetic relationship between strain HQ1 and related species based on the 16S rRNA gene sequences. Bootstrap values obtained with 1000 repetitions were indicted as *percentages* at all branches

16S rRNA sequence, was analysed with the Robust Phylogenetic tree online tool (Dereeper et al. 2008, 2010) and the neighbor-joining dendrogram was constructed (Fig. 1). Based on the dendrogram, morphological and biochemical characteristics, HQ1 was 80 % identical with *Ochrobactrum* sp. and as a result was tentatively identified as *Ochrobactrum* sp. HQ1. The nucleotide sequence encoding the 16S rRNA of *Ochrobactrum* sp. HQ1 (1121 bases) was deposited in the GenBank database with the accession number of KC577852.

### Growth rate of *Ochrobactrum* sp. HQ1 on 2-HQ

The growth experiment was performed in MSM fortified with concentration of 50 mg L^−1^ of 2-HQ. *Ochrobactrum* sp. HQ1 was grown on 2-HQ—amended minimal medium the cell count was enumerated at regular intervals (Table 1). The viable cell count immediately after incubation was recorded at 84 × 10^9^ CFU/mL. The total viable cell count increased to 158 × 10^9^ CFU/mL at the 6 h interval (log phase). The growth rate and generation time were, therefore, calculated as per the equation—*K* = log *N*_t_ − log *N*_o_/log 2 × *t* and recorded to be 0.71 h and 42.6 min/generation, respectively.

**Table 1:** Growth of *Ochrobactrum* sp. HQ1 on 2-HQ

Incubation time in h	*Ochrobactrum* sp. HQ1 growth in CFU/mL
0	84 × 10^9^
6	158 × 10^9^
12	160 × 10^9^
18	161 × 10^9^
24	163 × 10^9^

Growth of *Arthrobacter* sp. HY2 was observed after incubation in MSM containing 50 mg L^−1^ PNP in MSM after 6 h with concomitant decrease in PNP and was further increased by two folds within 12 h (Qiu et al. 2009). Similarly, commencement of growth of *Arthrobacter protophormiae* RKJ100 on PNP was noticeable after 2 h and reached maximum on 12–16 h (Chauhan et al. 2000; Ghosh et al. 2010). Optimum growth of *Serratia* sp. DS001 was found at a concentration of 0.3 mM PNP with doubling time of 13.8 h (Pakala et al. 2007). However, growth of *Ochrobactrum* sp. HQ1 on different metabolite—2-HQ has longer generation time.

### Biodegradation of 2-HQ by bacterial isolate

In order to investigate the influence of various environmental factors on the biodegradation of 2-HQ by *Ochrobactrum* sp. HQ1, the strain was cultured under different conditions and the biodegradation of 2-HQ analysed.

#### Influence of additional carbon source on biodegradation of 2-HQ

**Fig. 2:**
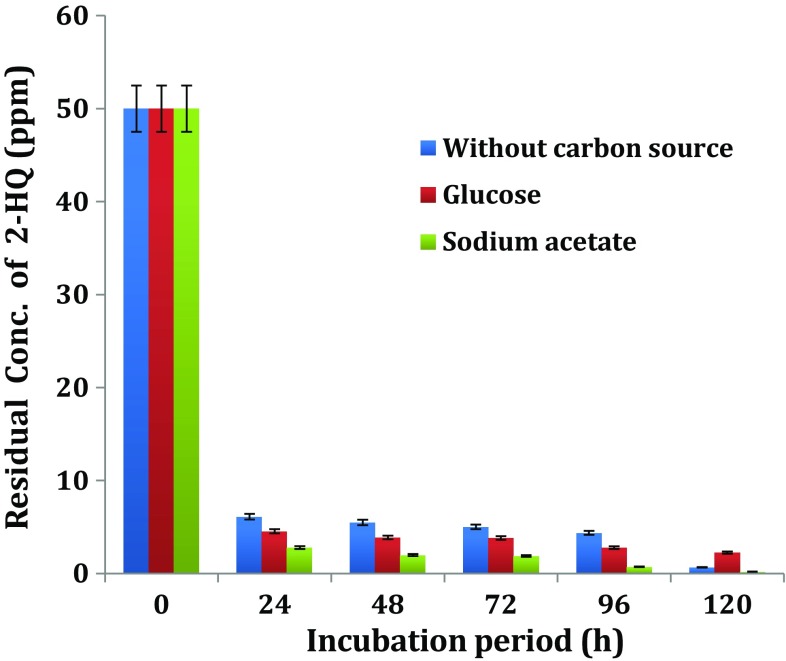
Influence of additional carbon source on biodegradation of 2-HQ

Biodegradation of 2-HQ by the bacterial isolate HQ1 in the presence or absence of glucose or sodium acetate in MSM was compared. The extent of 2-HQ disappearance was determined as presented in (Fig. 2).

Disappearance of 2-HQ in uninoculated medium with/without additional carbon was insignificant. However, about 90 % of added 2-HQ disappeared in HQ1 inoculated media supplemented with glucose or sodium acetate after 24 h of incubation. There was no significant difference in of the  % degradation of 2-HQ in the presence or absence of additional carbon. Therefore, it is clear from the results of the present study that the degradation of 2-HQ by *Ochrobactrum* sp. HQ1 bacterial strain was not influenced by the additional carbon.

In contrast with this results, addition of glucose (100 mg L^−1^) as co-substrate for EBN-12 mutant strain resulted in complete degradation of 100 mg L^−1^ of *p*-nitrophenol, a metabolite in degradation of parathion and methyl parathion in 20 h rather than in 24 h in its absence (Rehman et al. 2007). Addition of glucose at low concentrations (0.1–0.5 %) enhanced the degradation of PNP by *Pseudomonas* sp. (Schmidt et al. 1987) and *Arthrobacter* sp. HY2 (Qiu et al. 2009). This enhancement effect was not positively related to glucose concentration as higher degradation occurred at 0.1 % glucose than at 0.3 or 0.5 % glucose. Recent report by Reddy et al. (2014) was also in agreement with this observation. In the present study, only low concentration 0.01 % glucose was included in MSM but had no influence on degradation of 2-HQ by *Ochrobactrum* sp. HQ1.

#### Influence of additional nitrogen source on biodegradation of 2-HQ

The effect of organic (urea and yeast extract) and inorganic (ammonium chloride and sulphate) nitrogen sources on the HQ1 biodegradation of 2-HQ was compared to find the suitable source. The potential bacterial isolate—*Ochrobactrum* sp. HQ1, was grown on 2-HQ in MSM amended with the additional nitrogen source chosen from—inorganic forms—ammonium chloride and ammonium sulphate, organic forms—urea and yeast extract for biodegradation of 2-HQ. Appropriate controls—MSM devoid of both the additional nitrogen source and inoculum, MSM devoid of the additional nitrogen source with receipt of inoculum and inoculated MSM amended with additional nitrogen source were used.

**Fig. 3:**
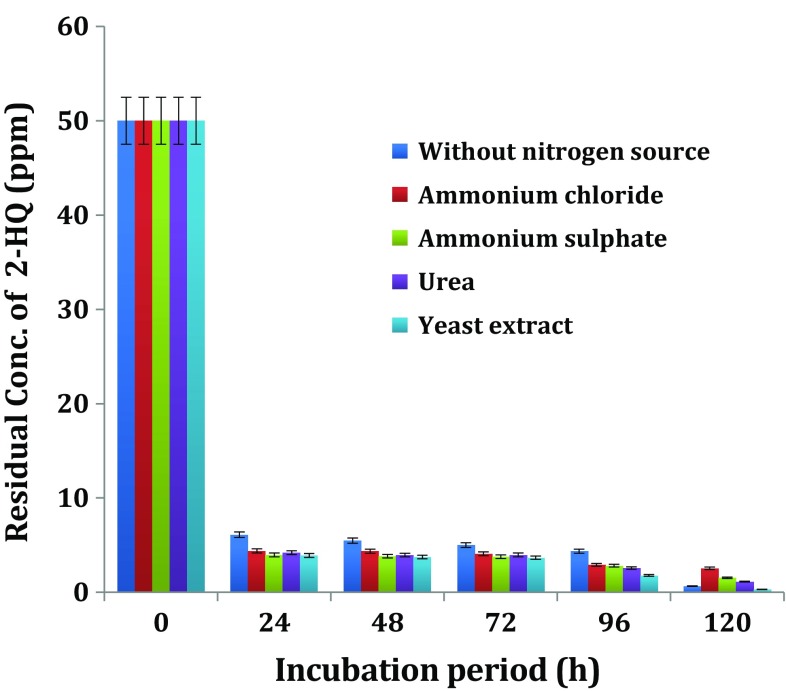
Influence of additional nitrogen source on biodegradation of 2-HQ

Degradation of 2-HQ occurred to the extent of 80–92 % in the culture of *Ochrobactrum* sp. HQ1 at the end of 1-day incubation (Fig. 3). Growth of *Ochrobactrum* sp. HQ1 on MSM without additional nitrogen resulted in 80 % degradation of 2-HQ compared to the 90–92 % degradation that was observed when the cells were grown on MSM with different additional nitrogen sources. Results of the present study indicate that the supplementation of MSM with yeast extract had a beneficial but marginal effect on degradation of 2-HQ by *Ochrobactrum* sp. HQ1.

In contrast to the results obtained in this study, Qiu et al. (2007) reported that addition of ammonium chloride and ammonium sulphate (1 g L^−1^) did not favour the growth of *Ochrobactrum* sp. B2 or degradation of parathion or methyl parathion metabolite—PNP. Addition of nitrogen sources (0.04 %) did not exert significant effect on PNP degradation (Kulkarni and Chaudhari 2006; Srilatha 2012). Quinoline is bicyclic aromatic compound and is structurally similar to 2-HQ due to presence of one benzene ring and one heterocyclic ring but differs from 2-HQ with one nitrogen in heterocyclic ring. Zhu et al. (2008) reported that additional nitrogen source in particular (NH_4_)_2_SO_4_ enhanced the growth and degradation of quinoline by *Rhodococcus* sp. QL2. In the present study, supplementation of additional nitrogen in the form of yeast extract marginally improved degradation of 2-HQ by *Ochrobactrum* sp. HQ1.

#### Influence of inoculum density on biodegradation of 2-HQ

**Fig. 4:**
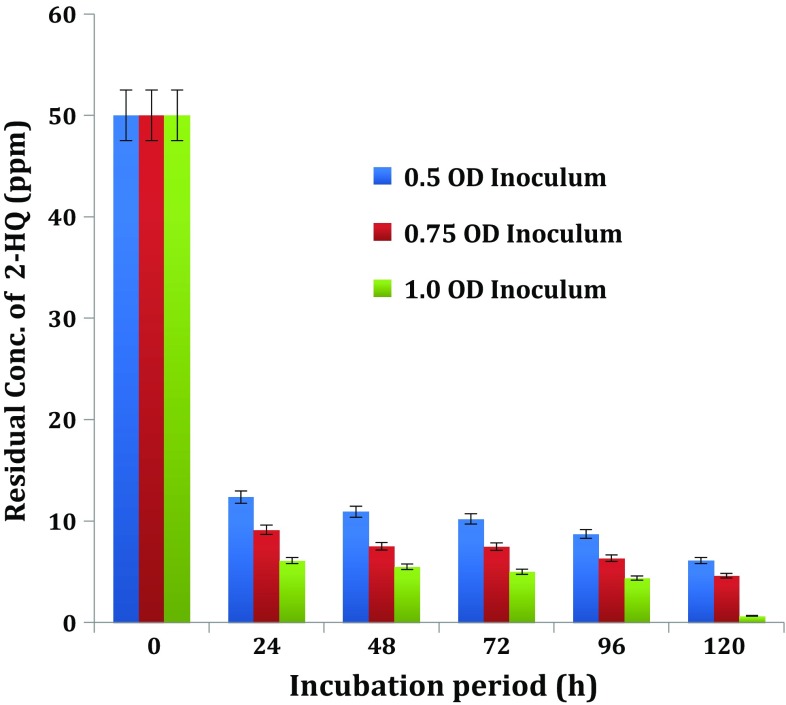
Influence of inoculum density on biodegradation of 2-HQ

In order to find out the optimal size of inoculum density for degradation of 2-HQ,—*Ochrobactrum* sp. HQ1 was cultivated on MSM after inoculation with the three different sizes of inoculum densities. The cell suspension of the bacterial culture was adjusted to the desired cell densities and added to MSM fortified with 2-HQ to provide initial cell densities in inoculated medium at 0.5, 0.75 and 1.0 OD 600 nm. The HQ1 cultures with initial cell density of 0.5, 0.75 and 1.0 OD, caused 2-HQ degradation to the extent of 75.28, 81.27 and 87.78 % at the end of 24 h incubation, respectively (Fig. 4). Thus, *Ochrobactrum* sp. HQ1 with highest initial cell density, i.e. 1.0 OD, supported the maximum degradation of 2-HQ indicating that the inoculum of 1.0 OD was the optimum cell density.

The results of the present study are in agreement with an observation in degradation of the parent compound methyl parathion and its metabolite PNP by *Pseudomonas cepacia* was high when large inoculum size was used (Keprasertsp et al. 2001). Labana et al. (2005) examined influence of cells/g soil (2 × 10^5^, 2 × 10^6^, 2 × 10^7^, 2 × 10^8^ and 2 × 10^9^) in the degradation of methylparathion metabolite—(PNP) by *Arthrobacter protophormiae* RKJ100. Among these inoculum densities, 2 × 10^5^, 2 × 10^6^ cells/g soil depleted PNP after 10 days of incubation, whereas 2 × 10^9^ cells were able to degrade PNP after 5 days of incubation. The optimum inoculum size was found to be 2 × 10^8^ which achieved 98 % depletion in just 2 days. The authors explained that when lower inoculum densities were used, the small number of bacteria was not able to survive the initial competition and population decline that usually occurs following inoculation.

#### Influence of concentration of 2-HQ on its biodegradation

*Ochrobactrum* sp. HQ1 bacterial isolate was grown on 2-HQ at different concentrations of 50, 100, 200 and 500 mg L^−1^ in MSM. Degradation of 2-HQ by the culture was examined and presented in the (Table 2). Concentration of 2-HQ had no influence on degradation of 2-HQ by *Ochrobactrum* sp. HQ1 culture.

**Table 2:** Influence of initial concentration of 2-HQ on degradation by *Ochrobactrum* sp. HQ1 under sub-merged culture conditions

Incubation period in h	Residual concentration of 2-HQ in MSM			
	50 ppm	100 ppm	200 ppm	500 ppm
0	50	100	200	500
24	6.11	8.25	19.71	25.61
48	5.49	5.04	18.21	25.56
72	5.01	3.59	17.91	25.00
96	4.37	3.02	11.78	24.66
120	0.66	1.89	7.54	9.00

Values presented in the table are means of triplicates

About 88–95 % of the added 2-HQ disappeared in the culture of *Ochrobactrum* sp. HQ1, irrespective of initial concentration present in the medium compared to the 1 % observed in controls at the end of 24 h incubation.

These results are consistent with the observed 90 % degradation of trichloro-2-pyridinol, a metabolite of chlorpyrifos by *B. pumilus* C2A1 at a high concentration of 300 µg mL^−1^ (Anwar et al. 2009). In a recent study, Reddy et al. (2014), reported on a *Bacillus* sp. that degraded 2-HQ at a high concentration of 500 µg mL^−1^. According to Yang et al. (2005), a strain of *Alcaligenes faecalis* strain DSP3 had the capacity to degrade wide range of TCP concentration from 10 to 800 mg L^−1^.

On the other hand, contrasting with this results, *Paracoccus* strain TRP degraded 3,5,6-trichloro-2-pyridinol (TCP) concentration of TCP 400 mg L^−1^ within 4 days (Xu et al. 2008). Recent report Lu et al. (2013) evidenced that a bacterial strain, *Cupriavidus* sp. DT-1 completely degraded TCP up to concentration of 50 mg L^−1^ within 14 h. Qiu et al. (2009) reported that concentration of degradation of PNP, a metabolite of methyl parathion, in the medium had influence on its degradation by *Arthrobacter* sp. HY2. More than 90 % PNP was depleted within 24, 48 and 168 h from the medium with initial concentration of PNP at <250, 350 and 400 mg L^−1^ of PNP, respectively. Very little degradation of PNP was detected at 450 mg L^−1^ after incubation for over 48 h. Virtually, PNP degradation was observed at a concentration of 500 mg L^−1^ of PNP for a period of 7 days.

Zhu et al. (2008) reported that the rate of degradation of *N*-heterocyclic aromatic compound—quinoline by *Rhodoccus* sp. QL2 increased with increasing concentration of quinoline up to 240 mg L^−1^ whereas rate decreased at higher quinoline concentration due to substrate inhibition.

#### Influence of medium pH on biodegradation of 2-HQ

**Fig. 5:**
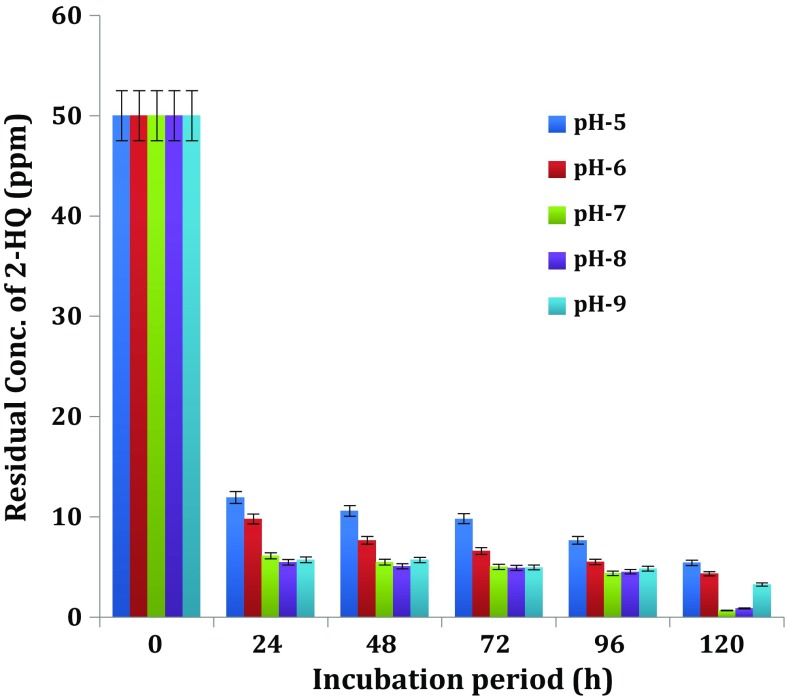
Influence of medium pH on biodegradation of 2-HQ

The pH is also an important environmental factor, which influences the growth of microorganisms, stability and solubility of enzymes and, in turn, their degrading ability of xenobiotic compounds. The 2-HQ degrading ability of potential bacterial isolate—*Ochrobactrum* sp. HQ1 was assessed during cultivation on 2-HQ-fortified MSM adjusted to, i.e. acidic 5, 6; neutral 7 and basic 8,9 (Fig. 5). The bacterial isolate—*Ochrobactrum* sp. HQ1 showed the degradation at all pH (acidic, neutral and basic) conditions tested.

Growth of *Ochrobactrum* sp. HQ1 at pHs 5, 6, 7, 8 and 9 caused 2-HQ degradation to the extent of 76.15, 80.41, 87.78, 89.08 and 88.58 % at the end of 24 h incubation, respectively. The per cent of disappearance of 2-HQ in respect of uninioculated control for each pH did not exceed 1 % of the added 2-HQ. The degradation was optimum at pH ranges 5–9 and 7–8, an observation that is consistent with reports by Reddy et al. (2014) on a *Bacillus* sp. that optimally degraded 2-HQ at pH range 6–8. Xu et al. (2008) reported that cell-free extracts of *Paracoccus* strain TRP strain degraded TCP in the pH range of 5–9, with the most rapid degradation rate at pH 8. According to Yang et al. (2005), *Alcaligenes faecalis* strain DSP3 capable of biodegradation of TCP required optimal pH of 8. The degradation rate was similar at pH 7 and 9, and slowest was observed at the two pH limits (6 and 11).

The greatest degradation of PNP by *Arthrobacter* sp. HY2 (Qiu et al. 2009) and by *Ochrobactrum* sp. B2 (Qiu et al. 2007) under slightly alkaline conditions (pH 7–9) was observed. Degradation of PNP required optimum pH range 7.5–9.5 for different bacteria (Kulkarni and Chaudhari 2006; Srilatha 2012; Labana et al. 2005; Unell et al. 2008; Wan et al. 2007). Maximum degradation of a *N*-heterocyclic aromatic compound—quinoline by *Rhodococcus* sp. QL2 and *Comamonas* sp. occurred at pH 8 (Zhu et al. 2008; Cui et al. 2004).

#### Influence of temperature on biodegradation of 2-HQ

**Fig. 6:**
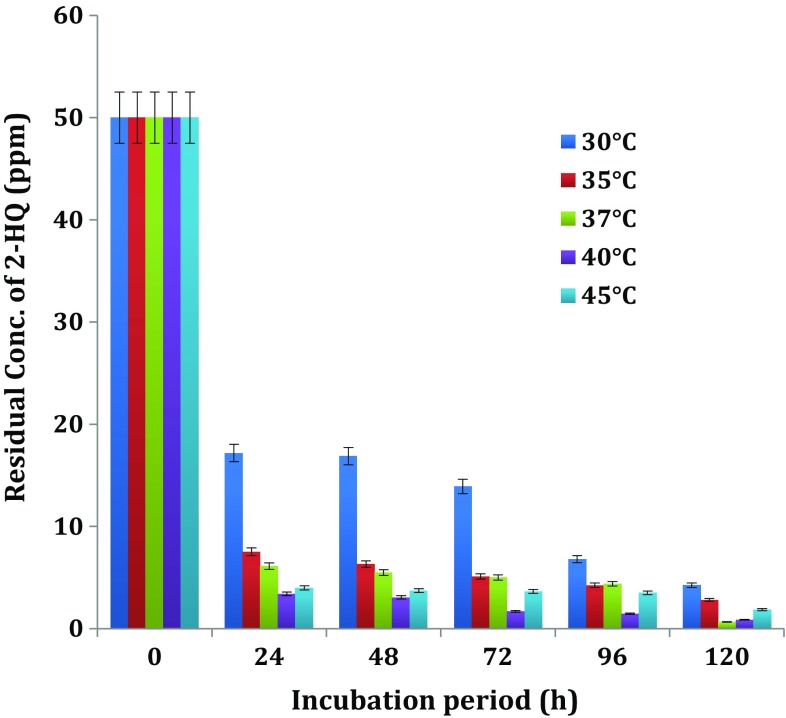
Influence of temperature on biodegradation of 2-HQ. Values presented in Figs. 2, 3, 4, 5, 6 are means of triplicates + deviation

In order to find out the optimum temperature for degradation of 2-HQ, by bacterial isolate *Ochrobactrum* sp. HQ1, culture was cultivated in MSM fortified with 50 mg L^−1^ of 2-HQ at different temperatures, i.e. 30, 35, 37, 40 and 45°C along with appropriate controls. The *Ochrobactrum* sp. HQ1 bacterial strain showed the degradation at all temperatures with varying proportions (Fig. 6).

Degradation of 2-HQ with 65.62, 80.96, 87.78, 93.2 and 92.02 % was observed in culture of *Ochrobactrum* sp. HQ1 grown at temperatures of 30, 35, 37, 40 and 45°C at the end of 24 h interval, respectively. The degradation of 2-HQ did not proceed beyond 1 % of added 2-HQ in uninoculated controls. It is clear from the results that the optimal temperature range from 37–40°C favoured the degradation of 2-HQ by *Ochrobactrum* sp. HQ1.

Mineralization of TCP by *Pseudomonas* sp. strain ATCC 700113 in liquid MSM at the concentration of 100 mg L^−1^ most rapidly occurred at a temperature of 28°C (Feng et al. 1997). According to Srilatha (2012), optimal temperature for degradation of PNP by the bacterial cultures *Arthrobacter* sp. and *Nocardioides* sp. ranged between 37 and 40°C. Degradation of PNP, a metabolite of methyl parathion by *Arthrobacter protophormiae* RKJ100 (Labana et al. 2005) in soil microcosms and *Arthrobacter* sp. HY2 (Qiu et al. 2009) indicated that degradation is most rapid at 30°C. The bacterial cell-free extracts of *Paracoccus* strain TRP strain degraded 3,5,6-trichloro-2-pyridinol (TCP), at temperature ranging from 15 to 40°C and the most rapid degradation rate was at 35°C reported by Xu et al. (2008). A strain of *Alcaligenes faecalis* strain DSP3 caused biodegradation of TCP most rapidly at 30°C (Yang et al. 2005). In a recent study (Reddy et al. 2014) observation of optimum temperatures of 37–45°C for the degradation of 2-HQ by *Bacillus* sp. was in confirmity with the result of the present study. The optimum temperature for degradation of quinoline by *Rhodococcus* sp. QL2 (Zhu et al. 2008) and *Comamonas* sp. (Cui et al. 2004) was found to be 35–40°C and 30°C, respectively.

#### Identification of 2-HQ metabolic intermediates through GC–MS analysis

In support of the data obtained from growth of bacterial isolates and HPLC analysis, further experiment was carried out to identify the metabolites, if any, formed during the degradation of 2-HQ by potential bacterial isolate *Ochrobactrum* sp. HQ1. MSM fortified with 2-HQ was cultivated with bacterial isolate under optimal conditions. Aliquots of the culture medium and uninoculated control were withdrawn at different time intervals and extracted with dichloromethane; samples were analysed through GC–MS as mentioned earlier in the “Detection and identification of 2-HQ metabolites by GC-MS analysis”. When degradation products extracted from the spent medium prepared from the 2-HQ supplemented culture were analysed by GC–MS with reference to the control, several characteristic peaks were obtained at different time intervals with different molecular ion [M^+^] at (m/z) values.

GC–MS analysis detected the presence of a metabolite with two different retention times of 6.44 and 18.67 with same mass of 281 in 24 h culture broth of *Ochrobactrum* sp. HQ1 grown on 2-HQ (Supplementary figures a, b).

**Fig. 7:**
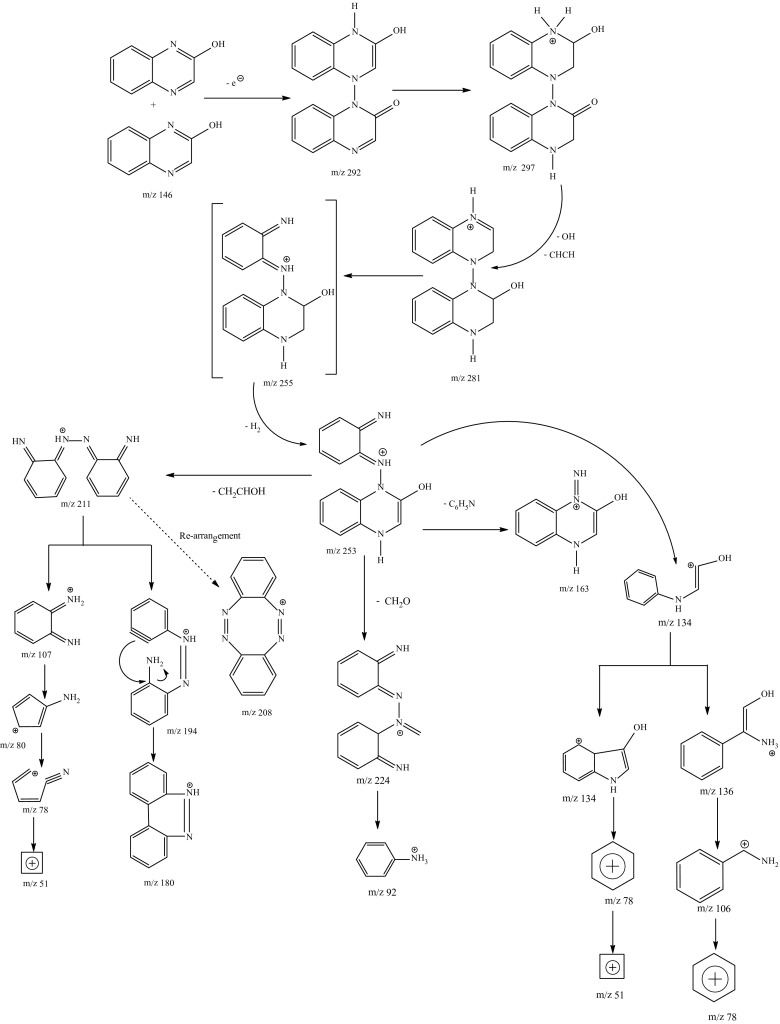
Tentative degradation pathway of 2-HQ by *Ochrobactrum* sp. HQ1

Formation of this metabolite would be expected only with dimerization and is tentatively identified as a derivative of dimer of 2-HQ shown in the Fig. 7. GC–MS analysis of 48 h culture broth of the same organism after extraction indicated the presence of another metabolite with retention time of 2.02 min and mass of 207 in addition to the above mentioned metabolite (Supplementary figure c). GC-MS analysis revealed the presence of metabolite with retention time of 14.47 min and mass of 281 in both 24-h and 72-h culture broth of *Ochrobactrum* sp. HQ1.But, pattern of fragmented ions derived from the metabolite in GC-MS spectra at the respective intervals was different (Supplementary figure d).

The metabolite with mass of 207 would be expected with opening of pyrazene ring in dimer metabolite. The same metabolites were also formed in the culture broth of other organism—*Bacillus* sp. Reddy et al. (2014). Formation of these two metabolites (m/z-281, 207) in microbial metabolism of 2-HQ appears to be the first report with gram-negative bacterial species to our knowledge and needs to be further confirmed with authentic standards. Based on these tentative metabolites and their fragmented ions, a pathway shown in the Fig. 7 is proposed for degradation of 2-HQ by *Ochrobactrum* sp. HQ1.

Generally metabolites, formed from xenobiotics during degradation, are identified (Kaur and Sud, 2012; Sutherland et al. 1996) with application of advanced tools like GC–MS. Using this approach, metabolites such as 4-nitrocatechol, 1,2,4-benzenetriol, hydroquinone and *p*-benzoquinone were identified in metabolism of *p*-nitrophenol by *Arthrobacter protophormiae* (Chauhan et al. 2000). Similarly, *p*-nitrophenol was identified as a degradative product of methyl parathion by *Serratia* sp. DS001 (Pakala et al. 2007). Recent report Lu et al. (2013) confirmed that with GC-MS analysis metabolites—3,5,6-trichloro-2-pyridinol (TCP) and 2-pyridinol were identified in degradation of chlorpyrifos by *Cupriavidus* sp. DT-1. Recently, Reddy et al. (2014) have reported that the same metabolites were formed in the metabolism of 2-HQ by gram-positive *Bacillus* sp.

## Conclusions

The bacterial isolate was identified to be *Ochrobactrum* sp. HQ1 through the selective culture enrichment method, morphological, biochemical and 16S rRNA sequence analyses. The optimum environmental conditions for growth and degradation of 2-HQ were analyzed in shaking conditions and recorded as the inoculum density of (1.OD), pH (7–8), 37–40°C temperature and high concentration of 2-HQ (500 ppm). This is the first report on degradation of 2-HQ by aerobic Gram negative bacterium and elucidation of pathway. Based on this pathway *Ochrobactrum* sp. HQ1 is the best remedial source in treatment of contaminated environment with pesticides and their metabolites.

## Electronic supplementary material

Below is the link to the electronic supplementary material.

Supplementary material 1 (DOCX 1195 kb)
