# Supplementary material for: Optimization study of 2-hydroxyquinoxaline (2-HQ) biodegradation by Ochrobactrum sp. HQ1
Source: 3 Biotech. 2016 Feb 8;6(1):51. doi: 10.1007/s13205-015-0358-6 (PMC4746200; doi:10.1007/s13205-015-0358-6)

**Supplementary figures:**


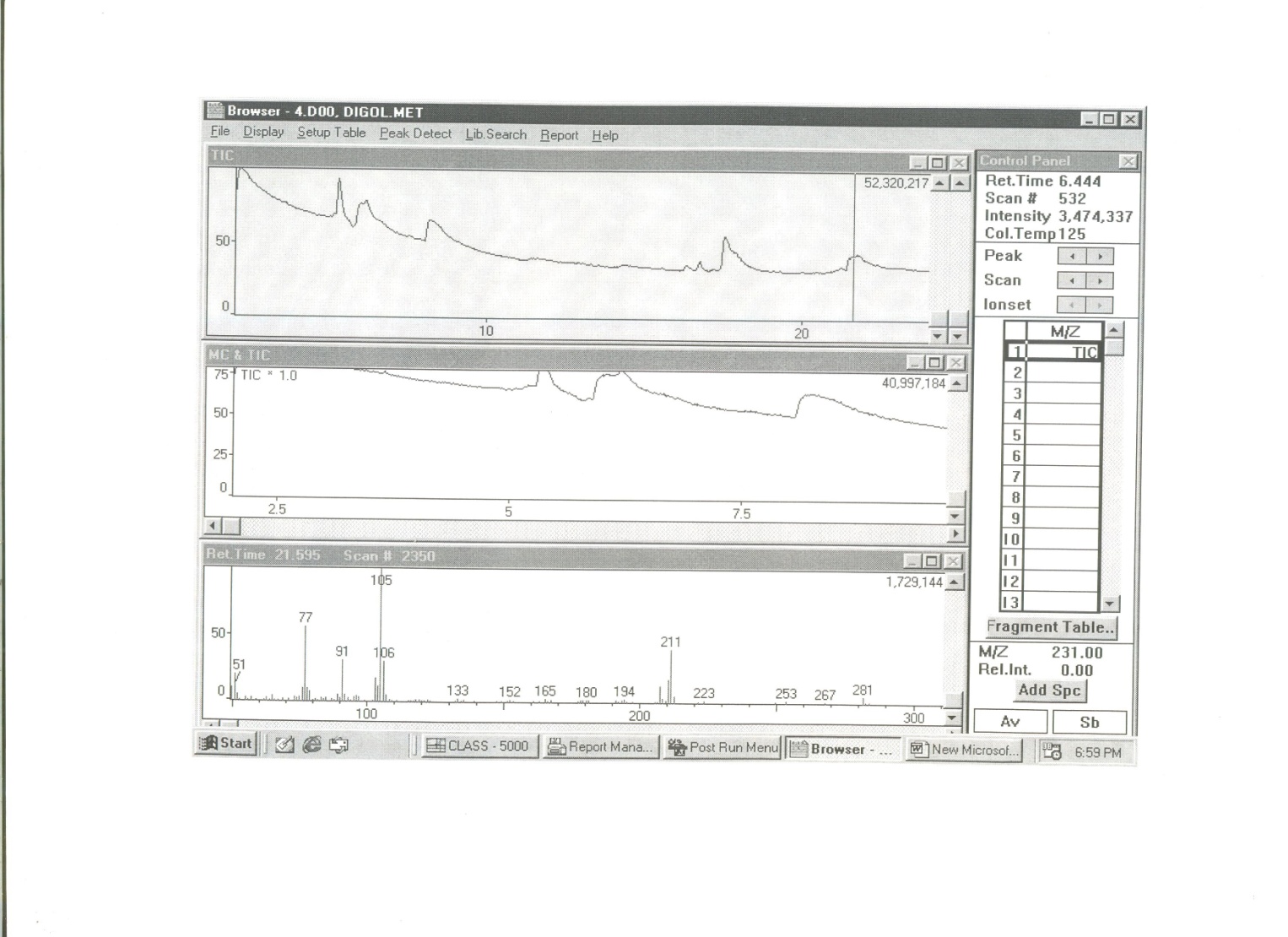
a. GC-MS chromatogram of 24-h culture broth of *Ochrobactrum* sp. HQ1 grown on 2-HQ (RT-6.44)

b. GC-MS chromatogram of 24-h culture broth of *Ochrobactrum* sp. HQ1 grown on 2-HQ (RT-18.67)


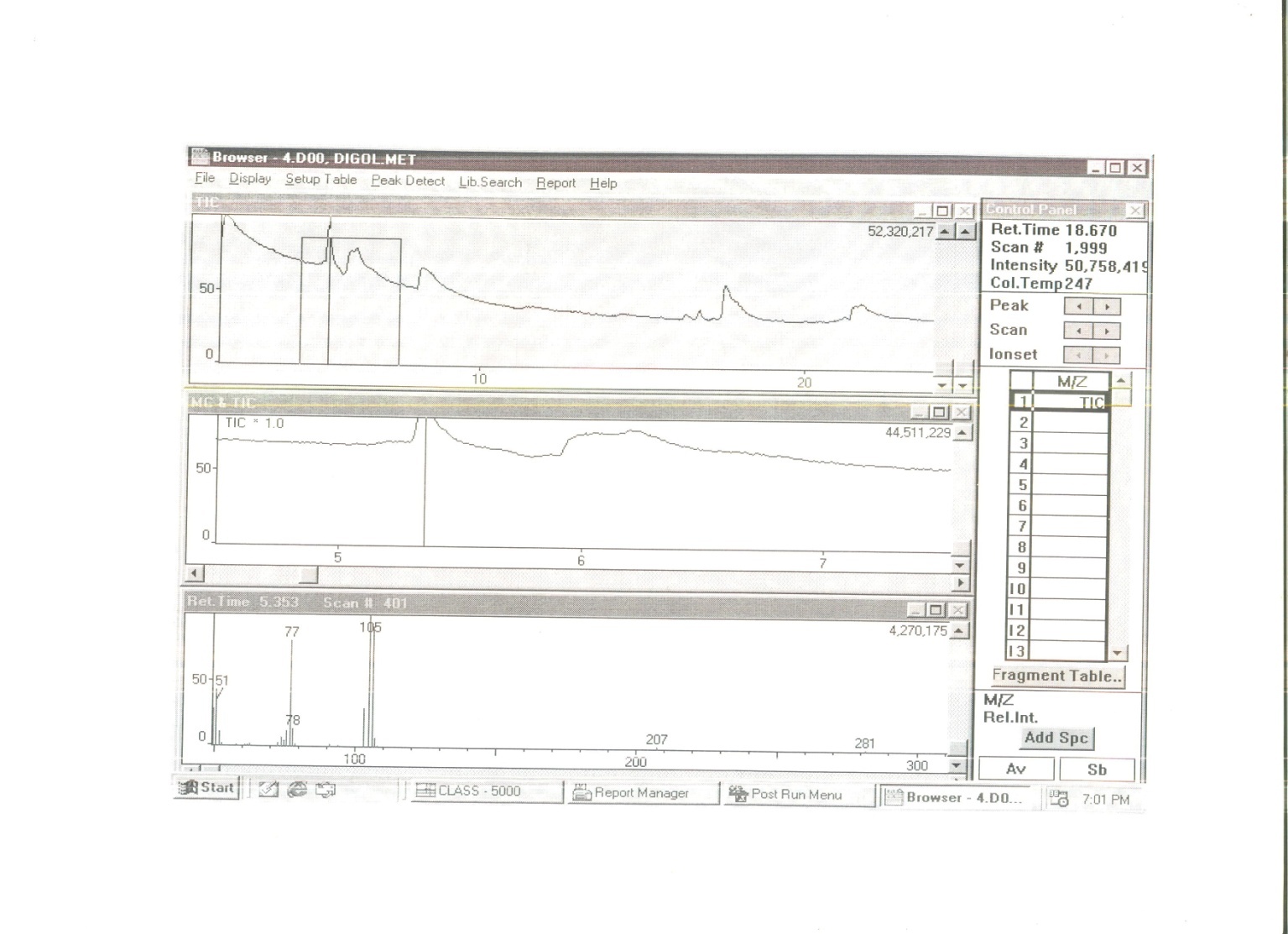


c. GC-MS chromatogram of 48-h culture broth of *Ochrobactrum* sp. HQ1 grown on 2-HQ


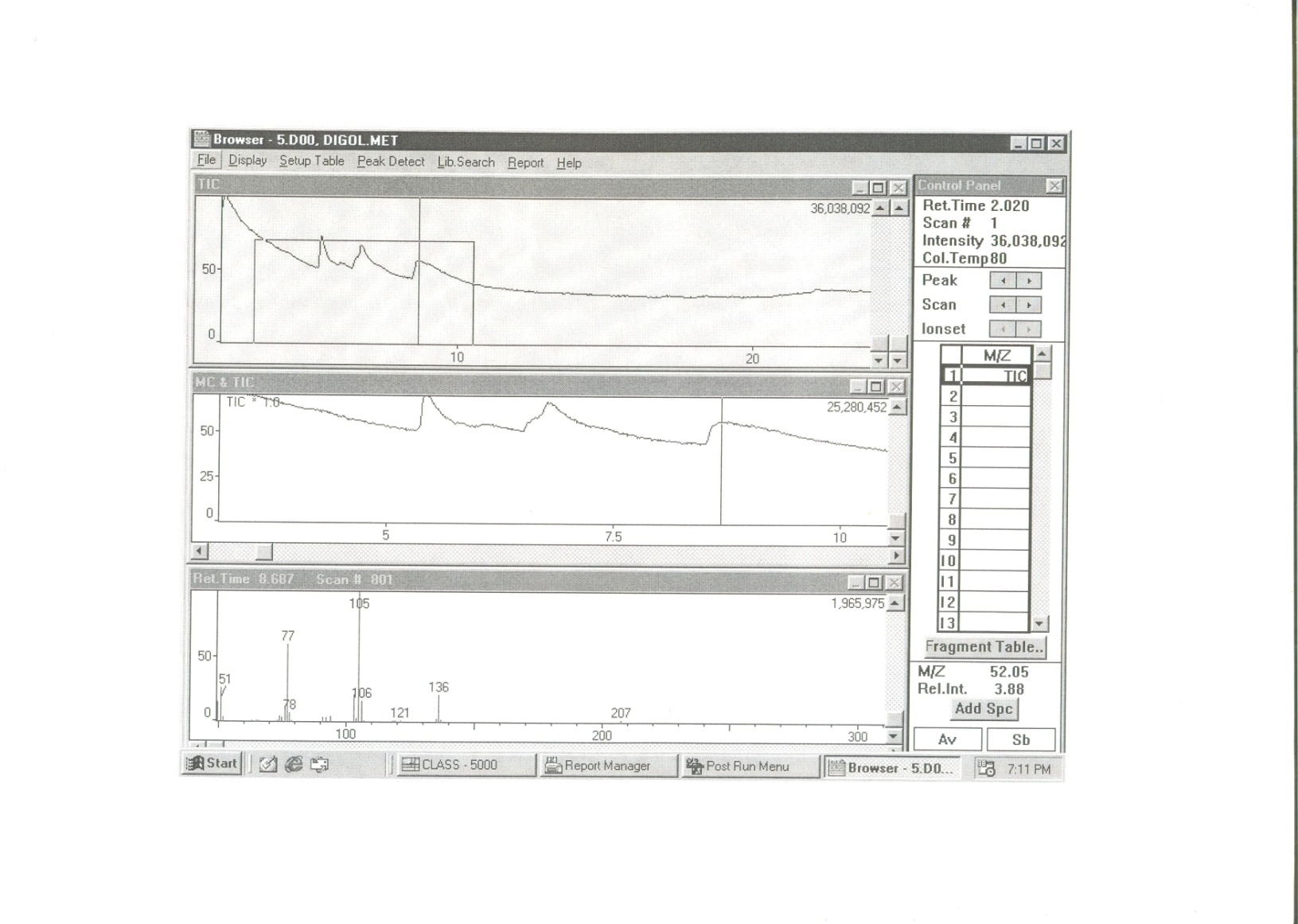


d. GC-MS chromatogram of 72-h culture broth of *Ochrobactrum* sp. HQ1 grown on 2-HQ


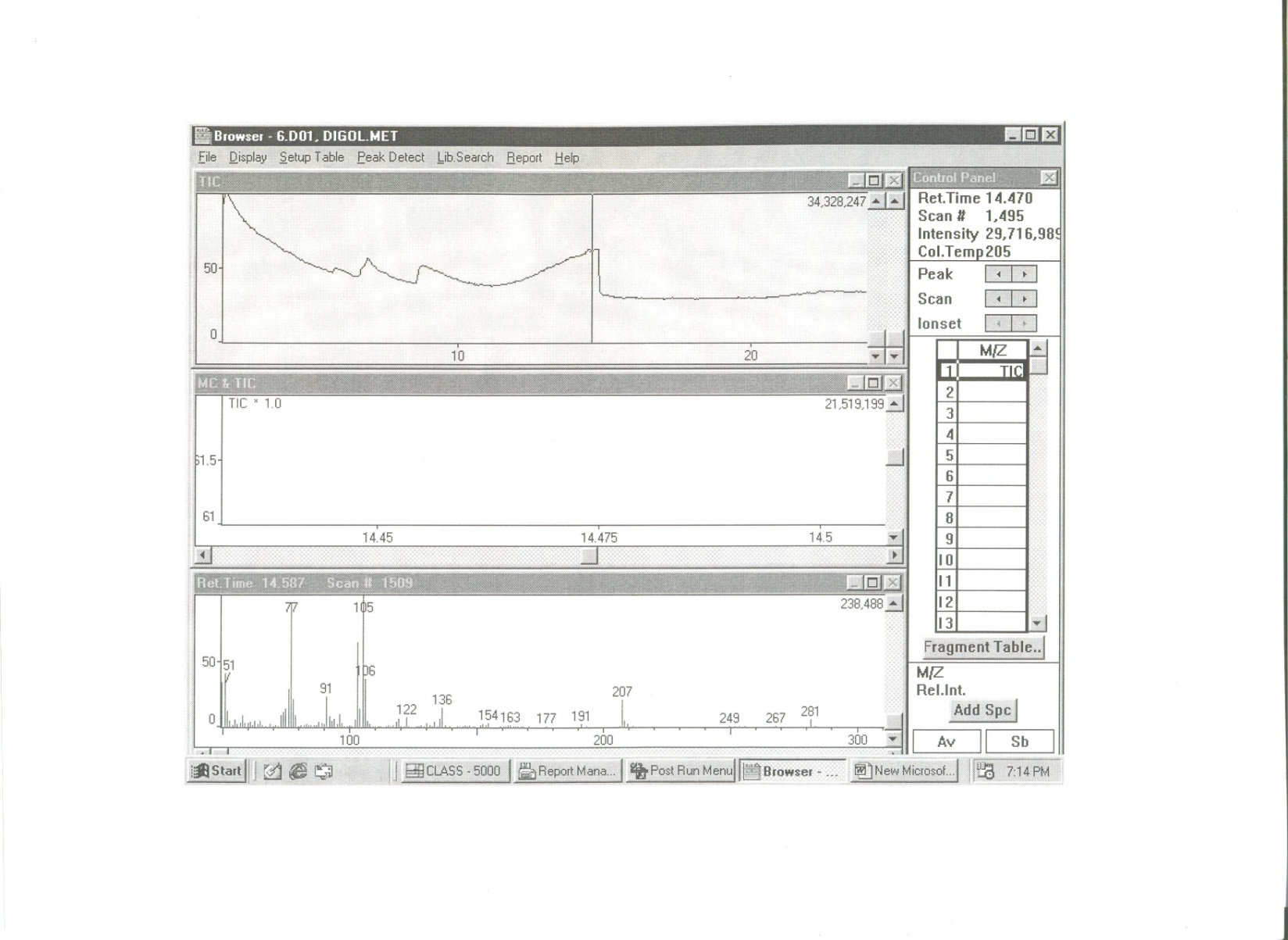

Supplement: Supplementary file 1 — Supplementary material 1 (DOCX 1195 kb) [file 13205_2015_358_MOESM1_ESM.docx]
